# Predicting human visuomotor behaviour in a driving task

**DOI:** 10.1098/rstb.2013.0044

**Published:** 2014-02-19

**Authors:** Leif Johnson, Brian Sullivan, Mary Hayhoe, Dana Ballard

**Affiliations:** 1 Department of Computer Science, University of Texas at Austin, TX, USA; 2 Department of Psychology, University of Texas at Austin, TX, USA; 3 Smith-Kettlewell Eye Research Institute, San Francisco, CA, USA

**Keywords:** visual attention, eye movements, reward, top-down control, state uncertainty

## Abstract

The sequential deployment of gaze to regions of interest is an integral part of human visual function. Owing to its central importance, decades of research have focused on predicting gaze locations, but there has been relatively little formal attempt to predict the temporal aspects of gaze deployment in natural multi-tasking situations. We approach this problem by decomposing complex visual behaviour into individual task modules that require independent sources of visual information for control, in order to model human gaze deployment on different task-relevant objects. We introduce a softmax barrier model for gaze selection that uses two key elements: a priority parameter that represents task importance per module, and noise estimates that allow modules to represent uncertainty about the state of task-relevant visual information. Comparisons with human gaze data gathered in a virtual driving environment show that the model closely approximates human performance.

## 1. Introduction and background

Human vision interrogates complex, noisy, dynamic environments to accomplish tasks in the world. For example, while driving a car, a person navigates to a desired destination (e.g. grocery store) while paying attention to different types of objects in the environment (pedestrians, vehicles, etc.) and obeying traffic laws (speed limit, stop signs, etc.). Humans manage these competing demands for visual information via the deployment of a foveated visual system, which must be actively moved to different targets to obtain high-resolution image information. How is this done, apparently so effortlessly, yet so reliably? What kind of a control structure is robust in the face of the varying nature of the visual world, and allows us to reliably arrive at our goals? Despite over a century of research on eye movements, we have very little knowledge of the way that the task demands of the visual world are handled by the brain. Most of the effort to formally model how gaze is deployed has focused on the properties of static images [1–3], but such models cannot account for task-based behaviour because they do not incorporate information about the goals and state of the agent whose vision is being modelled. Nonetheless, modelling task-directed vision during interactive natural behaviour in a three-dimensional world has been relatively unstudied, because it seems to require elaborate models of tasks.

Humans and other animals exhibit a variety of basic orientation and avoidance responses to visually salient stimuli, e.g. a looming stimulus can invoke avoidance. Presumably such basic responses were passed on over generations of animals that survived owing to the advantage provided. However, given the complexity of the world and the variety of rewards and punishments present, an attentional selection mechanism that is more elaborate than using image properties in a fixed stimulus–response relationship could prove useful. In particular, a learning mechanism that allows a mapping of the relationship between visual stimuli and task relevance, and a selection mechanism that can arbitrate between competing objectives that require visual information, would allow much more flexibility in behaviour during the life of an individual organism.

## 2. Modelling visual attention

The approach we take here is to consider vision as part of a control process where a human, or agent, actively chooses task-relevant information from the environment to guide actions and achieve goals [4–8]. Our visual task module approach is inspired by human visual behaviour, in particular a foveated visual system that can be highly specific in accessing particular pieces of information over time. Others have also suggested similar control theoretic or top-down driven approaches [5,9,10]. However, these models have not been applied to modelling human eye movements in a naturalistic, interactive three-dimensional setting. Our critical assumption is that complex behaviour can be broken down into simpler sub-tasks (which we refer to as modules), which operate independently of each other. This approach is suggested by observations of eye movements in natural behaviour, where subjects sequentially query the visual image for highly specific task-relevant information, and use that information selectively to accomplish a particular sub-task [11–13]. The problem then reduces to one of choosing which set of sub-tasks should receive new sensory information at any moment. One hypothesis is that sub-tasks are selected to be updated on the basis of a combination of both the behavioural importance of a specific goal, and the module's uncertainty about task-relevant visual information. This process can be formalized by using explicit representations of reward and uncertainty. This type of uncertainty-weighted reward decision framework was proposed by Sprague *et al.* [7]. They demonstrated a simulated agent that could successfully select task-relevant visual information coordinated among three competing tasks involved in navigation. However, this model was not applied to directly predict human gaze behaviour. This paper demonstrates a similar model of human visual processing and control where task-oriented modules representing reward and uncertainty are used to direct driving in a dynamic, noisy environment. Additionally, the model generates temporal variations in gaze deployment similar to those seen in humans driving in a virtual environment.

Each task module depends on its own set of world-state variables that are relevant to its specific task. In our model, we attempt to replicate the conditions of Sullivan *et al.* [14], and include modules for following a car, maintaining a constant speed and staying in a lane. In our driving simulation, relevant state variables for a car-following task module were the agent car's distance to a desired distance behind the leader and the difference between the agent's heading and the angle of this goal. The relevant state variable for a constant speed maintenance module was the absolute speed of the car. Finally, the lane module uses the car's angle to the nearest lane centre. These task modules run concurrently. However, to incorporate a foveation constraint, only one module at a time actively gains new perceptual information. While the human visual system is highly parallel, processing and attentional focus are largely biased towards the fovea, meaning humans typically get information in a serial fashion by foveating different objects over time. We assume that a consequence of this serial process is that when one visual task is accessing new information all other tasks must rely on noisy memory estimates. Based on averages from human data, fixation target selection calculations are repeated every 300 ms. In our data analysis, we accumulate multiple consecutive fixations onto the same object into ‘looks’, replicating the methodology of Sullivan *et al.* [14] (also discussed in [15]).

**Figure 1.**
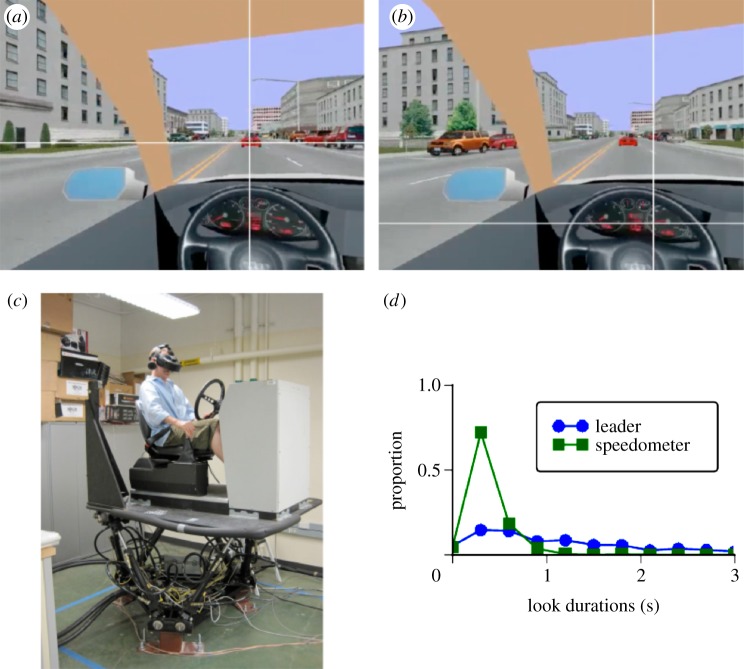
The dual task driving environment asks a subject to follow a leader car while maintaining speed. The commanded speed is slower than that of the leader car so the driver has to compromise, alternating checking between the speedometer reading and the position of the leader car. (*a*) A driver fixation on the leader car. (*b*) A driver fixation on the speedometer. (*c*) The car simulator used in the experiments. (*d*) A measured fixation duration histogram shows the distributions for the two tasks are very different. The speedometer can be read quickly, but following the leader car can require increased monitoring. (Online version in colour.)

Figure 1 depicts important elements of Sullivan *et al.*'s experiment relevant to our model. Subjects were instructed to drive lawfully and to successfully balance the two competing tasks of following a leader car (by maintaining a constant distance behind and staying in the same lane as the leader) and maintaining a constant prespecified speed. Figure 1*a,b* demonstrates examples of a human subject's fixating the leader car and speedometer, respectively. A photo of the simulator set-up and a subject wearing a head mounted display while driving is shown in figure 1*c*.

Sullivan *et al*.'s experimental conditions manipulated reward/priority for these tasks and varied the presence or the absence of noise in the speed maintenance task. This created scenarios where subjects needed to choose the most appropriate task to focus on at any given moment in time, e.g. if the reward is high for maintaining a speed, then the subject may select more information relevant to that task at the expense of task-relevant information for following. Figure 1*d* shows an example look duration histogram from the human dataset, demonstrating that human drivers devote unique amounts of time to the leader car and speedometer in this environment. Sullivan *et al*. designed the leader car's speed and the desired speed in the speed maintenance task to be dissimilar and conflict, forcing a decision on what visual information (leader car versus speedometer) would need to be fixated at any given time. Subjects were instructed to always perform both tasks, thus forcing the subject to make tradeoffs in selecting task-relevant objects for fixation. The task modules for our model were designed with the goal of mimicking these experimental conditions.

## 3. Driving simulation

The human subject's three-dimensional virtual driving world is simplified in the simulation as a two-dimensional plane. The simulated world contains a single road with two lanes similar to the one used by the human drivers. Our simulation has the option to generate other traffic in the environment; for reasons of simplicity, we did not include them in the work discussed here. A single non-agent car was designated as the leader car and followed a path with multiple lane switches similar to the one used in the human experiments. The basic simulation loop updates the state of the world at a 60 Hz frequency using an elementary physics simulation, to match conditions in Sullivan *et al.* Each module uses the formalism of a proportional-integral-differentiator (PID) to make a speed and and/or heading change that would direct the car towards a desired set point in the module's state space. At each time step, each car in the world moves ahead proportionally to the sum of the the combined recommendations of its speed and heading direction controllers, which can then be executed in the next simulated time step. Every time the simulator requests a control update, the modules are also updated by propagating time varying uncertainty estimates *σ*(*t*) according to each module's noise parameter (see appendix A for details).

If the simulation performed only the above steps, the agent's performance would become increasingly erratic over time, because the uncertainty estimates would drift further away from zero. The resulting erroneous state value estimates would produce poor PID controller outputs, and the resulting actions chosen by the agent would further compound the uncertainty in the state estimates. In a human driver, this behaviour would be analogous to taking one look at the world when getting into the car, and then driving with a blindfold thereafter. Clearly, this is not what humans do when driving. Instead, people continually and regularly reposition their gaze towards objects in the environment as their driving progresses. Thus, the final step in our model is to incorporate a scheduler that uses task uncertainty and priority to select a task module to receive new sensory information that can be used by the PID controllers. Like several others [6,5,16,17], we hypothesize that fixations serve to reduce uncertainty about the state of relevant variables in the world, in this case the distance and angle to the leader car, the agent car's current speed and lane position. To capture this behaviour, the simulator periodically selects one of these three modules for receiving updated state information through a perceptual arbitration mechanism.

## 4. The soft barrier model

**Figure 2.**
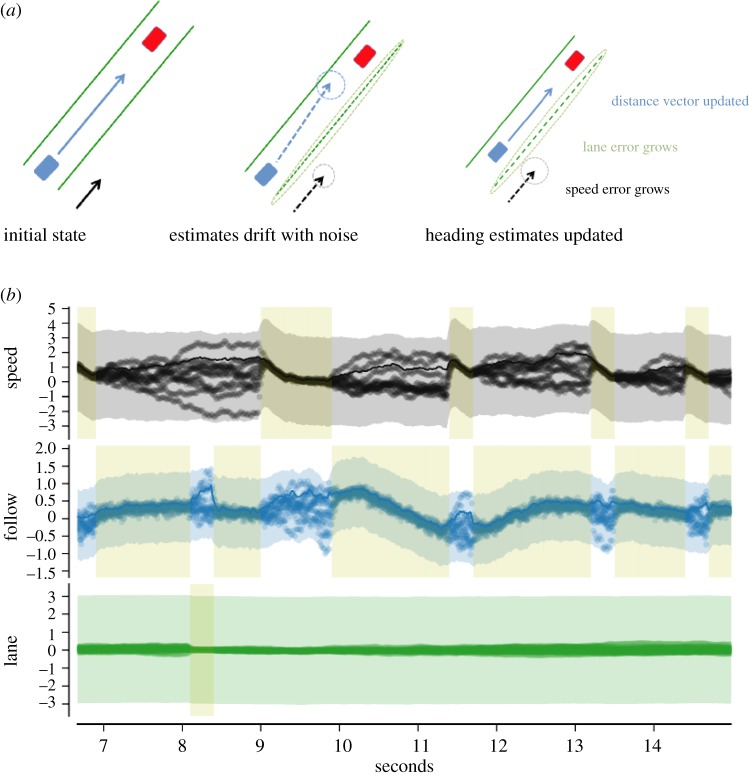
Evolution of state variable and uncertainty information for three single-variable modules. (*a*) Depiction of an update of the *Follow* module. Starting in the initial state, the necessary variables are known, but noise causes them to drift. According to the model probabilities, the *Follow* module is selected for a gaze induced update. This improves its state estimate while the other modules' state estimates drift further. (*b*) Depiction of the state estimates for the three modules: constant speed maintenance, leader following and lane following. In each, the line indicates a state estimate versus time for that module's relevant variable, in arbitrary units. Thus, for the speed module, the *y*-axis depicts the car's velocity, for the follow module it depicts the distance to a set point behind the lead car, and for the lane module it shows the angle to the closest lane centre. If estimates overlap into a single line, the module has low uncertainty in its estimate. If estimates diverge, making a ‘cloud’, the module has high uncertainty. An actual update from the simulation for the Follow module can be seen at 10 s. The fixation, indicated by pale shaded rectangle, lasts for 1.5 s. The figure shows how the individual state estimates drift between looks and how the state variables are updated during a look. The coloured transparent region shows *σ*(*t*) ± *ρ*(*t*) for each module. (Online version in colour.)

The perceptual arbitration process incorporates priority and uncertainty in the following way. We first define, for each module, a weighted uncertainty *ζ*(*t*) at time *t* as the difference between the RMS uncertainty *σ*(*t*) and the scalar priority *ρ*^(*i*)^. For module *i*,
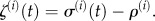


We also define a global variable *ϕ*(*t*) to represent the index of the module that gets updated at time *t*. We use a stochastic softmax decision mechanism in our module selection. The softmax principle [18] makes module selection probabilistic, transforming *ζ*(*t*) via a nonlinear sigmoid function. This increases the bias towards selecting high-priority modules and not selecting low-priority ones. However, the probabilistic nature of selection means that low-priority modules still have a non-zero chance of selection, which allows some flexibility in capturing the variability of human fixation behaviour. Diffusion to barrier (or boundary) models are used in modelling of decision-making in neural and human behaviour [19]. Our model incorporates elements of this decision rule by incorporating a noisy decision signal that evolves over time and uses a decision threshold. However, unlike those models, our model's threshold is probabilistic owing to the use of the softmax principle. Given the hybrid nature of our approach, we call it a soft barrier model. The soft barrier model [20] is defined as the probability that module *M*^(*i*)^ is selected for update at time *t*, using a Boltzmann distribution over each of the priority-weighted module uncertainties
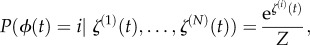
where *Z* normalizes the discrete distribution (see appendix A). This expression allows us to calculate the probability of the *i*th module being updated with new sensory information given our composite representation of priority and variance ζ across modules. Intuitively, if the uncertainty in *M*^(*i*)^ is currently above the threshold for that module—that is, if *σ*^(*i*)^(*t*) > *ρ*^(*i*)^—then *M*^(*i*)^ is much more likely to be selected for update than another module, especially if none of the other modules have uncertainties exceeding their thresholds. Note, in this form when the value of *ρ*^(*i*)^ is small, this translates into a high priority for that particular module, i.e. a low threshold means the variance for a module will contribute more to its chance of being selected. Figure 2 shows a caricature of a typical heading update as well as a typical segment of the performance of the three modules' boundary data and sensory update histories.

## 5. Model implementation and results

We implemented the model described above^1^ and ran several simulations to assess its behaviour. Our simulated driving environment was identical in layout to the virtual environment used by human subjects [14], in order to be able to directly compare our results to human performance. The implementation consisted of three modules: a ‘speed’ module *M*^(s)^ that attempted to drive at a particular target speed; a ‘follow’ module *M*^(f)^ that attempted to follow a lead car, and a ‘lane’ module *M*^(l)^ that attempted to steer so as to follow the nearest lane on the road. All cars in the simulation drove in a simulated two-dimensional world, described above. Each time gaze was allocated to a new module, we recorded the module that received the gaze, as well as several behavioural measurements (e.g. distance to leader car, current speed, etc.) to verify that the agent was driving appropriately.

**Figure 3.**
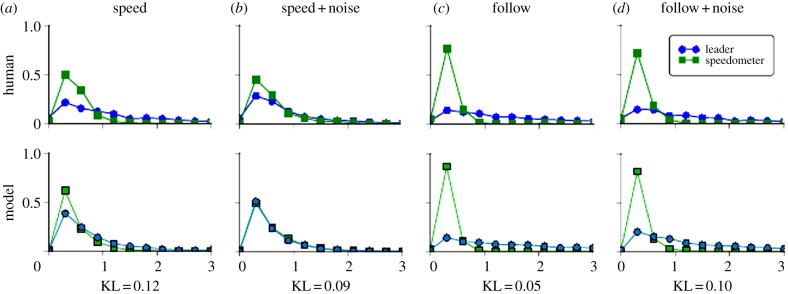
(*a*–*d*) Distribution of look durations for human subjects compared to the model predictions. In all plots, look duration (in seconds) is shown along the abscissa, with the proportion of looks indicated on the ordinate. Looks to the speedometer are plotted with squares; looks to the leader car are plotted with circles. In conditions where driving at a target speed was emphasized, human looks at the speedometer were approximately matched in duration to looks at the leader car. In conditions where following a leader car was emphasized, human looks at the speedometer were brief. Noise added to the car's speed affected human looks in the speed task more than looks in the following task. Similar results hold for our model. The KL divergences calculated between the human and model probability fixation density functions show that the model is in remarkably good agreement. (Online version in colour.)

Sullivan *et al.* [14] instructed subjects driving in a virtual environment to multi-task between the competing goals of trying to follow a leader car (at a distance of one car length and to follow any lane changes by the leader), and maintaining a constant speed. The priority of the two tasks was varied such that when one was high, the other was low, but but the subject must always attempt to do both and not simply ignore one of the tasks. Additionally, subjects drove in some conditions where noise was added to the speed of the car, with the intent of disrupting the maintenance of a constant speed. These manipulations resulted in four conditions where either following a leader or maintaining a constant speed was most important, and velocity noise was either present or absent. They found that task priority increased looks on task-related objects. Additionally, an interaction between priority and uncertainty was found, whereby uncertainty alone did not guarantee increased looks. Instead, only if a task-related object had sufficiently high priority did the addition of uncertainty further increase looks. Look duration histograms for this experiment are replicated in the top row of figure 3.

We ran a set of simulations with our model attempting to replicate this behaviour using parameters set to mimic the original human driver conditions. The model was able to capture several important aspects of the human data, including a sensitivity to both noise and priority, but also a gating effect whereby noise in low-priority tasks had a smaller effect than noise in high-priority tasks. Note, the methodology of Sullivan *et al*. counted looks on the road and task-irrelevant objects (i.e. looks that were not on cars or the speedometer) as a category termed ‘other’. As the majority of their analysis only addressed looks on the speedometer and on the leader car, we focus our presentation of results on these two modules.

Our model's results, shown under the row of human data in figure 3, are qualitatively similar to the human performance in a virtual driving environment. To compare these results more quantitatively, we used the Kullback–Leibler (KL) divergence [21], an information theoretic measure of the difference between two probability distributions measured in nats (the base *e* equivalent of bits). We calculated the individual human subject's divergence from the average human look duration distributions, and also calculated the divergence of simulations results with the average human distributions. The KL divergence values were averaged and found to be comparable: 1.06 for the human subjects versus 1.60 for the model.

In addition to our scheduling model, a noise-free fixation scheduler was run in the simulation. This scheduler incorporated only the priority of each task in selecting modules for update. Without the noise parameter, the KL divergence rose to 4.43, clearly demonstrating its importance to the model.

**Figure 4.**
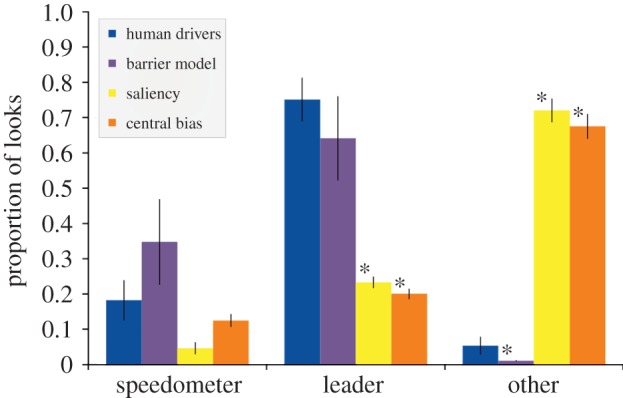
Comparing total fixations on task-relevant targets for three different models. Data averaged across the four conditions in Sullivan *et al*. [14] and all models. With respect to the speedometer task and the car-following task, the barrier model cannot be distinguished from the human fixation data. However, both the saliency and central bias models have very different allocations of fixations when compared with humans. Each have significantly less leader car fixations and significantly more ‘other’ fixations. Asterisks indicate statistically significant differences (*p* < 0.05) between a candidate model and the human driving data evaluated initially via ANOVA for main effects, with direct comparisons evaluated via student's *t*-test. (Online version in colour.)

To further emphasize the importance of task-based models, we compared the proportion of fixations on task-related targets using our model with two standard saliency measures, the Itti & Koch [1] saliency model and a central bias model [22]. Figure 4 shows these results and demonstrates how well the model fits the human data. By way of comparison, and as one might expect, without access to the particulars of the task, the observed data cannot be explained by the saliency or central bias models. Keep in mind that the model surely does not include all the aspects that the human is taking into account, but still is a very good approximation to the observed data.

## 6. Conclusion

Overall, the modular, soft barrier approach for modelling eye movements in human drivers provides an excellent account of fixation dynamics and suggests that task priority and uncertainty are primary controlling factors in allocating gaze in a multi-task situation. The computation of fixation choices depends only on these two parameters per module. Once they have been chosen, the prediction of gaze distributions is straightforward. A key component of our model arises from its compositional nature, which may scale to other, arbitrary multi-tasking situations given the appropriate selection and design of modules. Additionally, the softmax approach to selecting modules for sensory update has the useful mathematical property of being invertible. This means that human eye fixation data can be fed into the model and be used to provide the most likely set of parameter settings to explain those data. In our current simulations, we used a more simple approach to find reasonable parameter settings, but we plan to develop this inversion more fully and attempt to use this model to recover task priorities and uncertainty levels for the current human dataset as well as future experiments.

## Endnote

1 Source code at: http://github.com/lmjohns3/driving-simulator.

## Appendix A. Simulation details

### (a) Softmax selection

In definining *ϕ*(*t*), *Z* normalizes the discrete distribution, i.e. . Note, even if *ζ*^(*i*)^(*t*) > ζ^(*j*)^(*t*) for *j* ≠ *i*, there is some non-zero probability that *i* will not be selected for update at time *t*. Additionally, because module updates are always selected at frequency *f_p_*, by sampling from the above distribution at the appropriate time, a module might be selected for update even if none of the agent's task modules have exceeded their uncertainty boundary (i.e. if *ζ*^(*i*)^ < 0 for all *i*).

### (b) State uncertainty estimates

To incorporate this state uncertainty into the model, each module *M*^(*i*)^ maintains an explicit *estimate* of the current value of each of its state variables, *ŝ*^(*i*)^(*t*). (In the text for the sake of brevity, we omit the module superscript except where necessary to resolve ambiguities.) This estimate could be designed to incorporate many sorts of prior information about the dynamics of the world, but the model in its current state simply treats state estimates as independent and identically distributed samples drawn from a spherical normal distribution

where *μ*(*t*) = [*μ*_1_(*t*),…,*μ_n_*(*t*)]^T^ is a vector of the most recently observed state values, and *σ*(*t*) is the standard deviation for the state variable estimates in the module.

### (c) Uncertainty propagation

Uncertainty propagates over time within each module by maintaining a small set of *J* ‘uncertainty particles’  Each particle represents one potential path of deviation that the true state value might have taken from the last-observed state value. At every time step in the simulation, all uncertainty particles are displaced randomly by a step drawn from *N̂*(0,ε), thus defining a random walk for each particle. The RMS value of the uncertainty particles is then used to define the standard deviation

of state estimates for this module. Periodically, a module will be updated with accurate state information from the world (described below); when this happens, the magnitude of each uncertainty particle for the module is reduced according to *β_j_*(*t* + 1) = (1 − *α*)*β_j_*(*t*). After an informal parameter search, we set *α* = 0.7 for all modules; with *α* = 1, the model tends to produce many short updates because uncertainty is instantly reduced to 0, but with *α* < 1 the uncertainty increase due to noise competes with the uncertainty reduction from the updated state information.

The state estimation approach described here can be seen as a sort of particle filter [23], using an uninformed proposal distribution and equal weights for all particles. Interestingly, the behaviour of the simulation was largely unaffected by the choice of *J*; for our simulation, we used *J* = 10.

### (d) Priority

Modules can be prioritized by increasing their importance relative to one another, to allow modular agents to perform one task (for example, following a leader car) in preference to another (like achieving a target speed). In a traditional Markov decision process scenario [18], this is modelled by controlling the ratio of reward values between two subsets of world states. In this model, module priority is manipulated through the *ρ* parameter: as *ρ* increases, the module's relative priority decreases. This relative priority value is incorporated into the model as a soft bound on the diffusion of uncertainty for each module.

### (e) Simulation parameters

When searching for model parameters via grid search, owing to possible scaling ambiguities (e.g. if all *ε*^(*i*)^ and *ρ*^(*i*)^ are multiplied by 2, then the same qualitative behaviour will result) we fixed *ρ*^(f)^ = 1 and explored only settings for the other parameters.

### (f) Categorizing looks

The gaze selection process in our model is Markovian, meaning that each selected module is independent of the previously selected modules; more formally, *p*(*ϕ*(*t*)|*ϕ*(*t − n*),·) = *p*(*ϕ*(*t*)|·) for all *n* > 0. Thus, it is possible that multiple consecutive module updates are directed at the same module, or *ϕ*(*t*) = *ϕ*(*t − n*). Similar refixation behaviour exists in human gaze during complex tasks; presumably observers use the visual information across multiple fixations for a continuous control signal for a single task. To make analysis simpler and more consistent between simulation and human results, we grouped multiple consecutive updates for a given module into a single fixation.

Note, the data from Sullivan *et al.* presented in figure 4 omit data from the original including fixations on non-leader cars. Our model does not have a module devoted to gathering information from other cars, so these categories were ignored for all models.

### (g) Comparison with human results

We ran a set of simulations with our model attempting to replicate the human behaviour by fitting parameters set to mimic the orginal human driver conditions. Once we identified the parameter settings corresponding to the experimental conditions, we evaluated our model by running it in each of these conditions 10 times, with each simulation run for approximately 4000 steps. The sequence of module updates for each simulation run was stored and labelled as looks as described above, then normalized to form a probability distribution. These results were compared with the distributions of look durations from the human data. The model was able to capture several important aspects of the human data, including a sensitivity to both noise and priority, but also a gating effect whereby noise in low-priority tasks had a smaller effect than noise in high-priority tasks. In addition to our scheduling model, a baseline fixation scheduler was run in the simulation. This scheduler incorporated only the priority of each task in selecting modules for update, but uncertainty was not incorporated.

In comparing the overall fixation histograms for the different models, one-way ANOVA analyses were performed for each look category across human and model data. The proportion of speedometer looks was not significantly different between humans and the models, *F*_3,12_ = 2.4, *p* = 0.12. However, proportion of looks to the leader (*F*_3,12_ = 24.7, *p* < 1 × 10*^−^*^4^) and other category (*F*_3,12_ = 792, *p* < 1 × 10*^−^*^4^) were significantly different. For the leader and other category, pairwise comparisons were made; unpaired two-tailed *t*-tests were made comparing each model against human performance using Bonferonni correction for multiple comparisons. All *t*-tests compared the average of the mean subject/simulation performance across the four conditions for the leader and other object categories. Human data compared to central bias model were significantly different in both categories, leader: *t*_6_ = 8.6, *p* = 1.3 × 10*^−^*^4^, other: *t*_6_ = 39.2, *p* = 1.8 × 10*^−^*^8^; human data compared to the saliency model were significantly different in both categories, leader: *t*_6_ = 8.1, *p* = 1.9 × 10*^−^*^4^, other: *t*_6_ = 43.8, *p* = 9.4 × 10*^−^*^9^; lastly, human data compared to the barrier model only had a significant difference in the proportion of looks to the other category, leader: *t*_6_ = 0.22, *p* = 0.83, other: *t*_6_ = 7.4, *p* = 3 × 10*^−^*^4^.
